# Working Memory in Attention Deficit/Hyperactivity Disorder is Characterized by a Lack of Specialization of Brain Function

**DOI:** 10.1371/journal.pone.0027240

**Published:** 2011-11-10

**Authors:** Catherine Fassbender, Julie B. Schweitzer, Carlos R. Cortes, Malle A. Tagamets, T. Andrew Windsor, Gloria M. Reeves, Rao Gullapalli

**Affiliations:** 1 MIND Institute and Department of Psychiatry and Behavioral Sciences, University of California Davis, Davis, California, United States of America; 2 Maryland Psychiatric Research Center, Department of Psychiatry, University of Maryland, School of Medicine, Baltimore, Maryland, United States of America; 3 Division of Child and Adolescent Psychiatry, Department of Psychiatry, University of Maryland, School of Medicine, Baltimore, Maryland, United States of America; 4 Department of Radiology, University of Maryland, School of Medicine, Baltimore, Maryland, United States of America; The University of Melbourne, Australia

## Abstract

Working memory impairments are frequent in Attention Deficit/Hyperactivity Disorder (ADHD) and create problems along numerous functional dimensions. The present study utilized the Visual Serial Addition Task (VSAT) and functional magnetic resonance imaging (fMRI) to explore working memory processes in thirteen typically developing (TD) control and thirteen children with ADHD, Combined type. Analysis of Variance (ANOVA) was used to examine both main effects and interactions. Working memory-specific activity was found in TD children in the bilateral prefrontal cortex. In contrast the within-group map in ADHD did not reveal any working-memory specific regions. Main effects of condition suggested that the right middle frontal gyrus (BA6) and the right precuneus were engaged by both groups during working memory processing. Group differences were driven by significantly greater, non-working memory-specific, activation in the ADHD relative to TD group in the bilateral insula extending into basal ganglia and the medial prefrontal cortex. A region of interest analysis revealed a region in left middle frontal gyrus that was more active during working memory in TD controls. Thus, only the TD group appeared to display working memory-modulated brain activation. In conclusion, children with ADHD demonstrated reduced working memory task specific brain activation in comparison to their peers. These data suggest inefficiency in functional recruitment by individuals with ADHD represented by a poor match between task demands and appropriate levels of brain activity.

## Introduction

Impairments in working memory create difficulties for individuals with ADHD in their cognitive, academic and social activities. Difficulties arising from impaired working memory and associated executive functioning deficits may interfere with maintenance of rules to govern behavior, moral reasoning and problem solving skills [1]. Studies in both children and adults with ADHD suggest both verbal and spatial impairments in working memory [2], [3]. Discriminant and meta-analyses of working memory functioning in children and adults with Attention Deficit Hyperactivity Disorder (ADHD) [4], [5], [6], [7] suggest it is a prominent deficit associated with the disorder. Recent imaging studies on working memory in adults and children with ADHD have implicated a number of brain regions in ADHD working memory impairment, including frontal [8], [9], parietal [10] and temporal [11] cortices. Methylphenidate (MPH), a common treatment for ADHD, improves working memory performance in girls [12], boys [13] and men [14] with ADHD.

Working memory manipulation and maintenance is assumed to involve the prefrontal cortex (PFC) [15], [16], [17] and secondary brain regions, including the parietal lobe, to perform supporting processes such as verbal rehearsal, attention allocation and/or visual-spatial processing [18], [19]. Recent evidence suggests that PFC modulates top-down control biasing neural activity in posterior cortical regions [20], [21]. We hypothesize that altered PFC activity in ADHD [22], [23], [24], [25], [26] is accompanied by the recruitment of alternative regions that are ultimately less effective and flexible during working memory performance. Previous imaging studies examining working memory in adults with ADHD [14], [23], [27] and children [28], [29] suggest impaired PFC and anterior cingulate cortex (ACC) functioning, in conjunction with activation of a number of supplementary regions [30]. These regions include primarily posterior, inferior, and subcortical areas, suggesting recruitment of regions less traditionally associated with working memory functioning.

A core problem in ADHD beyond working memory processing is a failure to implement strategies and adjust brain activation to match particular demands, or modulate neural effort in response to task specific characteristics [26], [31], [32]. Recent data suggest that children with ADHD fail to effectively suppress activity in the default mode attention network with increasing cognitive demand, in comparison to typically developing peers. This impaired neural modulation is associated with an increase in RT variability [33]. ADHD may also be associated with the engagement of supplementary brain regions not traditionally associated with the task at hand [34], [35]. Indeed, one effect of stimulant medication in ADHD is that it appears to increase neuronal efficiency by increasing the signal to noise ratio during challenging tasks by reducing brain activity in non-task related regions (e.g [14], [36]).

This current study tested working memory in children with ADHD, Combined subtype, in comparison to TD children as an extension of previous neuroimaging working memory studies [14], [23] in adults with ADHD, Combined subtype. Both the previous adult and the present pediatric study employed a working memory task requiring overt manipulation of stimuli in a paced task. However, the present study utilized a paradigm with increasing task difficulty, which facilitates the investigation of neural modulation in response to increasing cognitive demand. We hypothesized that ADHD participants would exhibit 1) Diminished activity in prefrontal regions traditionally associated with working memory; 2) An excess in brain activation in regions associated with primary rather than higher cortical responding, including motor organization and output (i.e., basal ganglia); and 3) that brain activity in the ADHD group would not necessarily be modulated by increasing cognitive demand in a similar fashion to TD controls.

## Methods

### 1. Participants

Initial participants included 17 ADHD and 22 TD children between the ages 8 to 14 years. The final sample, selected to match the age, IQ, and SES of the ADHD group (see Table 1) included 13 ADHD and 13 TD children after six (four ADHD) were excluded due to excessive movement or requesting to discontinue the session. Participants taking stimulant medication (n = 9) did not take it for 48 hours before the fMRI session. The study included three left-handed participants, one in the TD group and two in the ADHD group. Participants received a $50 gift certificate and parents received $15/hour for their involvement.

**Table 1:** Demographics and Characteristics.

Variable	TD Group	ADHD Group
**Gender**		
Male	8	11
Female	5	2
**Ethnicity**		
Caucasian	9	11
African-American	3	1
Biracial	1	1
**Handedness**		
Right	11	12
Left	2	1

	Mean	Std. Dev.	Mean	Std. Dev.	t(24)	p
**Age**	10.6	1.8	10.7	4.13	0.08	0.94
**WISC-III Full Scale IQ**	117.62	12.15	117.23	10.69	0.09	0.93
**WJ-III Calculation**	113.77	10.97	113.08	13.6	0.14	0.89
**CPRS-R:L Scales**						
Cognitive Problems/Inattention	44.46	4.08	70.46	8.03	10.41	0.0001*
Hyperactivity	44.62	2.29	70.46	7.77	11.5	0.0001*
ADHD Index	43	2.58	72.23	5.95	16.26	0.0001*
DSM IV Inattentive	43.15	2.79	72.92	6.26	15.65	0.0001*
DSM IV Hyperactive	44.54	3.82	70.77	8.21	10.45	0.0001*
DSM IV Total	43.69	3.84	73.77	5.73	15.72	0.0001*
**CTRS–R:S Scales:**						
Cognitive Problems/Inattention	48.64	5.56	53.82	7.32	1.87	0.08
Hyperactivity	45.3	1.57	58.64	13.6	3.23	0.009*
ADHD Index	48.82	3.22	59.64	13.49	2.83	0.02*

*Note:* WISC: Wechsler Intelligence Scale for Children III Edition; WJ-III: Woodcock Johnson III Edition; CPRS-R:L: Conner's Parent Rating Scale-Revised: Long Version; CTRS-R:S: Conner's Teacher Rating Scale-Revised: Short Version; TD: Typical Developing.

Recruitment strategies included advertising in newspapers, pediatric and ADHD clinics, support groups and websites. Parents gave written informed consent, participants 13 and older gave written assent and younger participants gave verbal assent for a protocol approved by the institution's review board.

We only invited volunteers for the ADHD group if the participants met DSM-IV-TR criteria for ADHD, Combined Type to enhance our ability to identify brain activation alterations associated with individuals experiencing the combination of inattentive and hyperactive/impulsive symptoms. The ADHD diagnosis was based on the presence of ADHD via the Diagnostic Interview for Children and Adolescents (DICA, [37]), a follow-up clinical interview [38] and a score of 1.5 *SD* above the mean on the Total Scale of the DSM-IV ADHD Parent Conners' Rating Scale – Long Version (CPRS-R-L) [39]. Participants in the TD group did not meet criteria for ADHD on the DICA or follow-up clinical interview, and had T-scores below 60 on the DSM-IV ADHD CPRS-R-L Total Scale. The Conners' Teacher Rating Scale – Long Version (CTRS-R-L) [39] provided additional information on diagnostic status (see Table 1). Participants demonstrated the prerequisite calculation skills for the imaging paradigm via scoring within one *SD* or higher on WJ-III Calculation subtest for their age. All volunteers participated in all phases of the screening process.

Participants with co-existing Axis I or II diagnoses (except for ADHD in the ADHD group), metal or prosthesis in the body or major medical conditions were excluded. Children with first-degree family members with a history of bipolar, schizophrenia, or obsessive-compulsive disorder and controls with first-degree family members with ADHD were excluded. Exclusions were identified via phone screens that reviewed inclusion and exclusion criteria; the DICA and a follow-up interview with a clinician regarding clinical conditions in the participants and family members; Conners' Rating Scales; medical history, metal and prosthesis questionnaire and a handedness scale modified for children [40]. The Wechsler Intelligence Scale for Children – Third Edition (WISC-III; [41]) evaluated participants' intellectual abilities and the Woodcock-Johnson Tests of Achievement – Third Edition (WJ-III; [42]) was used to help assess learning disabilities. Evidence of a math/reading disorder was considered present if there was a 1.5 SD or greater difference between IQ and WJ-III scores. Assistants with a master's level degree in psychology or higher conducted the psychological testing and interviewing. A licensed, Ph.D. level psychologist reviewed all evaluation information to determine eligibility for the study.

### 2. Procedures

#### 2.1. Experimental Task

We developed the Visual Serial Addition Task (VSAT) based on the Paced Auditory Serial Addition Task (PASAT, [43]), as an fMRI-compatible, working memory task for pediatric populations. The PASAT produces working memory-related activation in adults with ADHD [14], [23], and is sensitive to stimulant treatment in children [44] and adults [14]. To validate the VSAT, we administered it to a larger sample of ADHD and TD controls [45] demonstrating that children with ADHD produce significantly more omission errors coupled with a higher variability in response time (RT) compared to TD. Task performance also correlated with Conners' ADHD Ratings.

**Figure 1:**
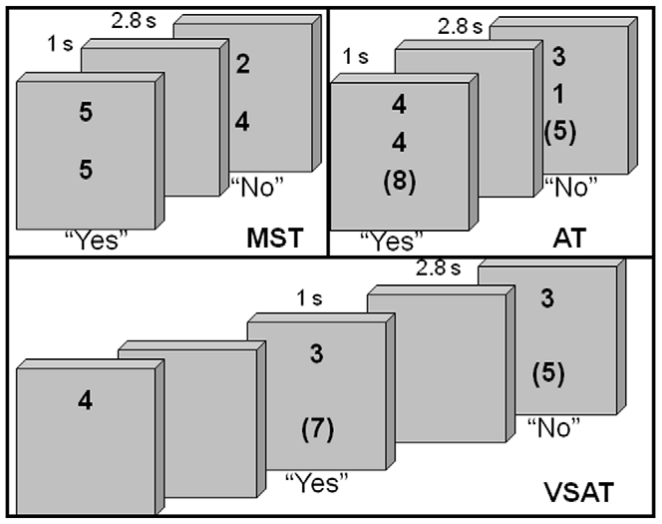
Paradigms. A) Match to sample task (MST). Subjects responded “yes” by button press if two numbers matched. B) Addition Task (AT). Subjects responded “yes” if the number in parentheses matched the sum of the numbers presented on screen. C) Visual Serial Attention Task (VSAT). Subjects responded “yes” if the sum of the number on screen and the number from the previous screen matched the number in parentheses.

The VSAT presents single-digit random numbers with participants adding each number to the one on the preceding screen. The stimuli ranged from 1–7 and could sum to no more than 9. (See Figure 1) Participants are required to compare the sum of these two numbers to the solution which is presented on-screen in parentheses. Participants respond “yes” if the sum held in memory matches the solution presented or “no” if it does not match the solution, with buttons in their right and left hands, respectively. Stimuli are composed of 60% correct and 40% incorrect answers. Due to the effect of the timing aspects of the PASAT [44], and VSAT on performance, a block fMRI design was used rather than an event-related design. Only participants performing a minimum of 85% correct in a previous practice session outside the scanner participated in the imaging session. This minimized the potential for disproportionate numbers of commission errors between groups contaminating or biasing group difference activation maps [46].

Two tasks controlled for different levels of cognitive involvement. A match-to-sample task (MST), with its less demanding cognitive load [47], was used as a task to control for sensory-motor demands of both the VSAT as well as an addition task (AT) while focusing the subject on a specific cognitive operation. During the MST, participants viewed two numbers presented in a vertical array on-screen. They were required to respond “yes” if the numbers matched and “no” via button press if they did not. A more demanding task, the AT, controlled for arithmetic ability, recall of simple math facts, motor and visual processing, and moderate cognitive processing demands. During the AT, two numbers and their “solution” (in parentheses) were presented in a vertical array on-screen. Participants responded yes if the sum of the two numbers presented matched the solution presented on the screen and no if they did not. Minimal working memory was involved in either control task as stimuli and answers were presented on-screen simultaneously. Task stimuli were presented for 1 sec with an ISI of 2.8 sec. Each task consisted of 10 events (an additional 4 MST events were included in the first block to allow for T1 equilibration effects) and one run of the paradigm alternated as follows: MST, AT, VSAT, MST, AT, VSAT, MST, AT, VSAT, MST. Each participant completed three runs of the task.

Independent samples t-tests examined whether there were any differences between the TD and ADHD groups on IQ, achievement and behavioral measures or ADHD ratings. Repeated Measures ANOVA with Task (MST, AT, VSAT) as a within-subjects condition and Group (TD, ADHD) as a between-subjects condition tested whether behavioral measures (percent correct, percent error, correct RT and omission errors) would be affected by increasing task difficulty from the MST to AT to VSAT tasks. We have previously demonstrated an increase in intra-individual RT variability in all subjects with increasing task difficulty in this paradigm [33].

#### 2.2. fMRI Data Acquisition and Analysis

A 1.5T Philips Eclipse scanner (Philips Medical Systems, Cleveland) equipped with high performance gradients acquired 88 high-resolution T1-weighted axial slices (TR = 25 msec; TE = 4 msec; matrix size = 256×256; 1.5 mm slice thickness; FOV = 230 mm). For five (three ADHD) participants, high-resolution structural scans were not acquired due to the child's request to terminate the scanning session early. Each functional run acquired 223 volumes (22 axial slices, 5 mm slice thickness, 1 mm gap) using single-shot, T2* weighted, echo-planar imaging sequences (TR = 2000 msec, TE = 35 msec, matrix size = 128×128, FOV = 230 mm). Vision 2000 goggles from Resonance Technologies (Northridge, CA) were used to present the stimuli.

Data were preprocessed and analyzed using AFNI software [48], (http://afni.nimh.nih.gov/afni). Volumes were motion corrected and all images aligned to the eighth volume in the run acquired immediately preceding the structural scan. The first seven volumes as well as volumes displaying excessive motion (more than one voxel size or 5 mm) were excluded from further analysis. Data were smoothed (4 mm Gaussian FWHM) and converted to percent change scores using MST as the baseline and any activation outside the brain was set to zero.

For each participant, data from all runs were concatenated together. Due to excessive movement, one run was not included for five ADHD children. To address this issue, analyses were conducted with matched runs between groups. To this end, we randomly chose 5 TD children and excluded their third run before conducting the contrasts. Ideal waveforms were created for AT and VSAT tasks by convolving a square-wave function with a hemodynamic response function; therefore, the MST task acted as the absolute baseline against which AT and VSAT activity was contrasted. Multiple regression analyses generated percent signal change for AT and VSAT relative to the MST baseline. Motion parameters were modeled as variables of no interest. Images corresponding to estimates of the parameters of interest were then warped into the standard Talairach space [49] (1×1×1 mm^3^).

Analysis of variance procedures for repeated measures were used to analyze the data in a 2×2 mixed design with group as a between-subjects factor (ADHD vs. TD), and condition as a within-subjects factor (AT vs. VSAT). Post-hoc contrasts included within-group (VSAT versus AT) and between-group (TD versus ADHD) contrasts. Significance required a voxel-wise threshold of p≤0.005. As a correction for multiple comparisons we combined this voxel-wise threshold with a minimum cluster-size of 282 µl, determined by Monte Carlo simulations, resulting in an overall 0.05 probability of a significant cluster surviving by chance. As our primary interest for this study is working memory functioning, we focus on relevant contrasts of interest, that is, post-hoc within- and between-group comparisons on the VSAT task (i.e., TD VSAT vs. AT; ADHD VSAT vs. AT; TD VSAT vs. ADHD VSAT), resulting from the ANOVA. Activation maps were thresholded and corrected for multiple comparisons as described previously.

To test our hypotheses of frontal hypoactivity in the ADHD group, we used a mask comprised of inferior and middle frontal gyri (MFG) and performed a region of interest (ROI) analysis. This mask was defined using a plugin within AFNI which defines specific anatomical regions of interest in Talairach space. We used small volume correction [50] to maintain the overall p value at 0.05 and determined any regions of activation within this area in the between-groups working memory contrast.

The average activation, per region, from each map (a. TD VSAT vs. AT, b. ADHD VSAT vs. AT and c. TD VSAT vs. ADHD VSAT) was calculated for each participant for each task (AT and VSAT). Bivariate correlation analyses tested the relationship between working memory task performance (correct RT) and activation within notable regions as defined by the ANOVA. Correlations also tested for a relationship between brain activity and omission errors in the ADHD group only as the TD did not exhibit sufficient variability in this measure.

Additional whole-brain regressions tested for correlations between RT and activation in every voxel in the brain in both groups separately. Within the ADHD group, whole-brain regressions tested the relationship between brain activation and symptom severity from the Hyperactivity, Inattentiveness and Total ADHD score of the CPRS-L. Again, activation maps were thresholded and corrected for multiple comparisons as described previously.

Analyses were conducted to ascertain whether gender or handedness inequality between the groups might have affected our results. These analyses revealed no significant differences in activation maps controlling for gender and handedness that were not explained by decreased power due to fewer participants per group. Therefore, we report only results including extra number of females and left-handed participants.

## Results

### 3. Behavioral Results

**Figure 2:**
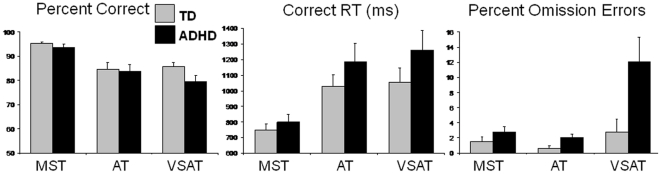
Behavior across the MST, AT and VSAT tasks. Both TD and ADHD groups displayed poorer performance across the three tasks used; there were less percent correct responses and slower RT with increasing difficulty. When examining omission errors, the TD group made relatively few errors in all three tasks. The ADHD group, however, did make more omission errors moving from the MST to the AT to the VSAT. Omission errors are considered to be a behavioral index of inattention.

Independent t-tests revealed that the groups did not differ across age, SES, or IQ (see Table 1). There were no differences in accuracy or RT between the ADHD and TD groups on the VSAT, AT or MST. Repeated measures ANOVA revealed main effects of Task (MST, AT, VSAT) on percent correct (F(1,24) = 29.65, p<0.0001), correct RT (F(1,24) = 52.33, p<0.0001) and percent commission errors (F(1,24) = 23.47, p<0.0001). Percent correct responses decreased from MST to AT to VSAT tasks, RT increased and commission errors increased. This suggests that all subjects displayed task-related impairments in their behavioral performance with increasing task difficulty (see Figure 2). With regard to omission errors, there was a main effect of Task (F(1,24) = 7.8, p = 0.001) and a Task×Group interaction (F(1,24) = 4.49, p = 0.02). As Figure 2 demonstrates, although the TD group made a minimal number of errors across all three tasks, omission errors in the ADHD group tended to increase with increasing task difficulty.

Independent t-tests revealed that the minimally-demanding MST resulted in no significant differences in response time, omission or commission errors between ADHD and TD groups. The ADHD group produced significantly more omission errors on the VSAT and AT paradigm, suggesting greater inattention on the more cognitively demanding tasks. Commission errors on the tasks did not differ between groups (see Table 2).

**Table 2:** Behavioral Performance.

Variable	TD Group		ADHD Group			
	Mean	Std. Dev.	Mean	Std. Dev.	t(24)	p
**VSAT**						
% Accuracy	87.6	6.66	83.26	8.89	1.41	0.17
Correct Response RT (msec)	1053.59	330.54	1259.92	459.31	1.32	0.2
% Incorrect Responses	12.21	6.7	15.68	8.5	1.16	0.26
Incorrect Response RT (msec)	1101.48	351.28	1321.57	373.89	1.55	0.14
Omissions	1.11	2.49	4.83	4.68	2.53	0.02*
**AT**						
% Accuracy	85.9	10.06	85.32	9.36	0.15	0.88
Correct Response RT (msec)	1027.74	273.94	1186.6	424.74	1.13	0.27
% Incorrect Responses	14.02	10	14.32	9.07	0.08	0.94
Incorrect Response RT (msec)	970.52	265.11	1067.21	527.9	0.57	0.57
Omissions	0.6	1.17	2.01	1.85	2.33	0.03*
**MST**						
% Accuracy	95.94	2.38	94.71	4.29	0.91	0.38
Correct Response RT (msec)	747.46	151.53	799.62	184.88	0.79	0.44
% Incorrect Responses	4.03	2.35	5.22	4.24	0.89	0.38
Incorrect Response RT (msec)	697.03	172.42	785.8	179.12	1.26	0.22
Omissions	0.67	0.99	1.21	1.2	1.24	0.23

*Note*: VSAT: Visual Serial Addition Task; AT: Addition Task; MST: Match-to-Sample Task.

Examination of the motion parameters (in six planes), using repeated measures ANOVA, revealed no significant between-group differences in the mean motion (Group Main Effect: *F*(1,24) = 3.19; *P* = 0.09).

### 4. fMRI Results

#### 4.1. ANOVA Main Effects and Interaction Effect

The main effect of Condition (AT, VSAT) revealed significant activation in thirteen regions including the PFC (BA 9 and 6), ACC, basal ganglia, temporo-parietal junction, inferior parietal cortex, precuneus, post-central gyrus and middle occipital gyrus (see Table 3A). The main effect of Group (TD, ADHD) revealed five regions including the left inferior frontal gyrus (IFG) extending into the insula (BA 46, 45 and 13), bilateral basal ganglia and right fusiform gyrus (see Table 3B). Finally, the Condition×Group Interaction revealed two regions, one in the left ACC and one in the right pre-central gyrus (see Table 3C).

**Table 3:** ANOVA Main Effects.

Region	Brodmann Area	Hem	Volume	Talairach coords. (centre of mass)		
			(µl)	x	y	z
				(RL)	(AP)	(IS)
***A) Condition***						
**Frontal lobes**						
SFG/medial FG/ACC	9/32	R	4773	7	−43	27
medial FG	6	R	585	10	9	61
**Basal Gaglia**						
Putamen		R	392	29	14	−3
Claustrum		L	387	−36	18	5
**Temporal lobes**						
MTG	39	R	1152	40	57	18
STG	22	L	335	−50	18	5
temporo-parietal junction	40/41/13	R	3684	48	25	23
**Parietal lobe**						
IPL	40	L	797	−49	33	25
Precuneus/postCG	4	R	429	9	33	57
Precuneus/post cingulate	31	L	1439	−13	57	14
**Occipital lobes**						
MOG/lingual gyrus	19	R	4671	27	71	6
MOG	18	L	1198	−28	82	8
Fusiform	19	L	511	−38	68	−14
***B) Group***						
**Frontal lobes**						
IFG/Insula/Claustrum	45/13	R	1284	25	−23	2
IFG/Insula	46/13	L	1176	−34	−22	16
**Basal Ganglia**						
Claustrum/insula	13	R	833	26	−6	11
Lentiform nucleus		L	499	−20	11	−4
**Occipital Lobes**						
Fusiform gyrus	37	R	373	40	54	−7
**C) Group×Cond**						
ACC	32	L	677	−15	−27	24
PreCG	6	R	301	35	17	35

*Note*: Hem., hemisphere; coords., coordinates; L, left; R, right; ACC, anterior cingulate cortex; IFG, inferior frontal gyrus; IPL, inferior parietal lobe; MTG, middle temporal gyrus; MOG, middle occipital gyrus; preCG, pre-central gyrus; postCG, post-central gyrus; SFG, superior frontal gyrus; STG, superior temporal gyrus.

For x, y, z coordinates, R, A & S are positive. corrected for multiple comparisons.

#### 4.2. WM-Related Contrasts of Interest: Within-Group Contrast in the TD Group

**Figure 3:**
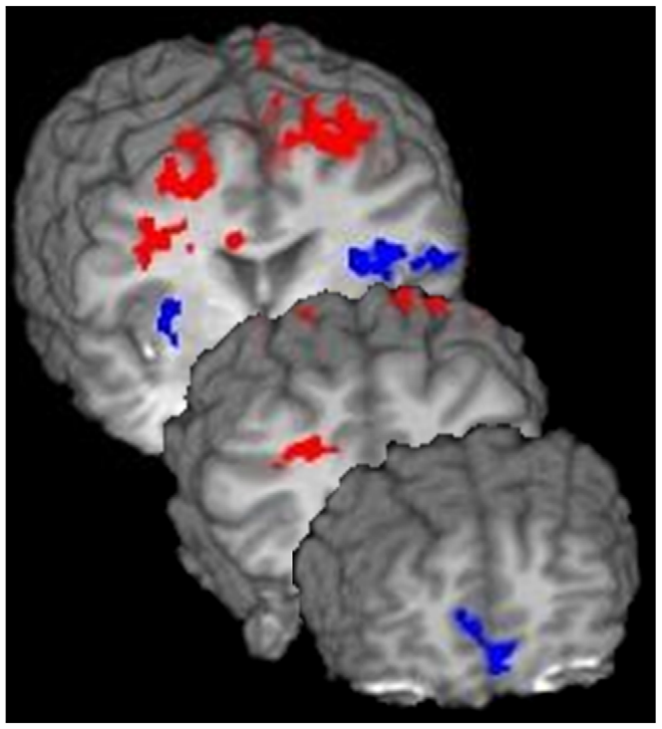
Regions from the TD Within-Group Contrast Map. TD controls activated bilateral frontal cortical regions, including MFG, and the cingulate gyrus during the working memory task (in red). Areas of deactivation during the VSAT included medial PFC and bilateral temporal lobes.

The TD within-group contrast map resulted in eighteen functionally-defined regions, including the MFG (BA 6 and 9), pre-central gyrus, ventro-medial PFC, putamen, and the temporal and parietal cortices (see Table 4A). Six of these regions were more active during the VSAT compared to the AT (see Figure 3.), namely the bilateral MFG (BA 6) and left BA 9, ACC, left pre-central gyrus, cingulate and bilateral post-central gyrus (BA 4). Pearson correlations including all participants revealed that faster RT on correct VSAT trials was associated with more activity in the left (r(26) = −0.39, p = 0.053) and right MFG (r(26) = −0.38, p = 0.05). Correlations within each group separately revealed that in the ADHD group alone, greater activity in the left (r(13) = −0.7, p = 0.007) and right MFG (r(13) = −0.54, p = 0.054) as well as the right cingulate (r(13) = −0.59, p = 0.04) correlated with fewer omission errors. Furthermore, there was a negative correlation between ADHD symptoms and activity in the bilateral MFG. Specifically, greater activity in the left MFG was associated with both fewer hyperactive/impulsive symptoms (r(13) = −0.44, p = 0.02) and a smaller ADHD Conners' Index score (r(13) = −0.41, p = 0.04) and the right MFG activity was associated with fewer hyperactive/impulsive symptoms (r(13) = −0.44, p = 0.03) on the Conners'. There was a trend for inattentive symptoms and Conners' ADHD DSM-IV Total score to correlate with left MFG activity (r(13) = −0.38, p = 0.06 and r(13) = −0.38, p = 0.06, respectively).

**Table 4:** WM-Related Contrasts of Interest.

Region	Brodmann Area	Hem	Volume	Talairach coords. (centre of mass)		
			(µl)	x	y	z
				(RL)	(AP)	(IS)
***A) TD VSAT v AT***						
MFG	6	L	5088	−25	2	47
	6	R	4869	18	5	57
ventro-medial PFC	32/10	L	1635	−1	−43	−1
MFG/ACC	9/32	L	436	−21	−24	27
PreCG	6	R	282	36	10	43
Cingulate	23	B	1721	0	16	25
Insula	13	R	1314	42	18	8
	13	R	1098	37	0	15
posterior cing	30	R	985	−14	58	13
Putamen		R	455	−31	−2	3
STG	38	L	471	41	−8	−14
MTG	21	R	291	56	11	−7
IPL	40	R	290	53	28	30
PostCG	4	R	400	15	30	67
	4	L	372	−18	29	54
Cuneus		R	2608	22	74	7
		L	1090	−23	82	23
IOG	18	L	2198	−32	80	−6
***B) ADHD VSAT v AT***						
medial FG		R	3375	11	−43	26
ACC	32	L	538	−12	−29	21
insula	13	R	300	32	21	12
STG	22	R	903	42	54	16
	22	L	469	−52	17	6
IPL	40	R	1814	49	26	25
	40	L	436	−49	26	22
IPL/supramarginal gyrus	40	L	285	−56	37	31
MOG/lingual gyrus	19	R	1964	29	69	3
***C) TD v ADHD VSAT***						
Claustrum/IFG/insula		R	1001	26	−23	2
Claustrum/insula/putamen	13	R	881	30	5	12
insula	13	L	502	−34	−20	18
medial FG	10	L	395	−12	−50	14
**D) ROI TD v ADHD VSAT**						
MFG	6	L	188	−24	10	53

*Note*: IOG, inferior occipital gyrus; PFC, prefrontal cortex; All other abbreviations as Table 3. Corrected for multiple comparisons.

#### 4.3. WM-Related Contrasts of Interest: Within-Group Contrast in the ADHD group

The ADHD within-group contrast map revealed nine regions, all more active in the AT compared to VSAT. These regions included the medial FG, occipital, parietal and temporal regions (see Table 4B).

#### 4.4. WM-Related Contrasts of Interest: Between-Group WM Contrast

**Figure 4:**
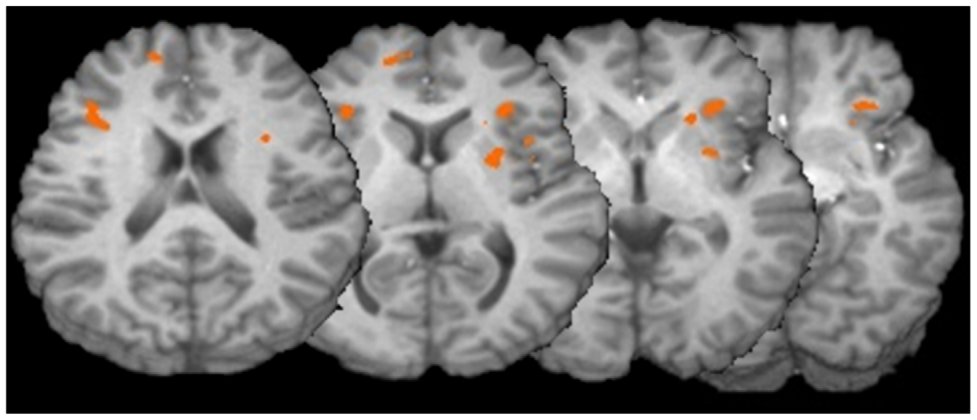
Regions from the Between-Groups Contrast Map. The between-group analysis revealed two regions in the right insula/claustrum one extending into the IFG and the other into the putamen, left insula and the left medial FG. All of these regions were more active in the ADHD compared to TD group.

The TD VSAT vs. ADHD VSAT contrast revealed four regions all significantly more active in the ADHD compared to the TD control group, namely two regions in the right insula/claustrum, one extending into inferior frontal gyrus (IFG) and the other into putamen, left insula and left medial frontal gyrus (see Figure 4 and Table 4C). Pearson correlations revealed a positive correlation between activity in the right claustrum/putamen and RT to correct responses on the VSAT (r(26) = 0.48, p = 0.01) and left insula and correct VSAT RT (r(26) = 0.40, p = 0.04) such that more activity was associated with longer RT. These correlations did not reach significance when we examined both group separately (right claustrum/putamen p = 0.07 in the ADHD group). Correlations with ADHD symptoms in the ADHD group alone revealed that the DSM-IV Total ADHD score correlated positively with activity in the left insula (r(13) = 0.60, p = 0.03).

**Figure 5:**
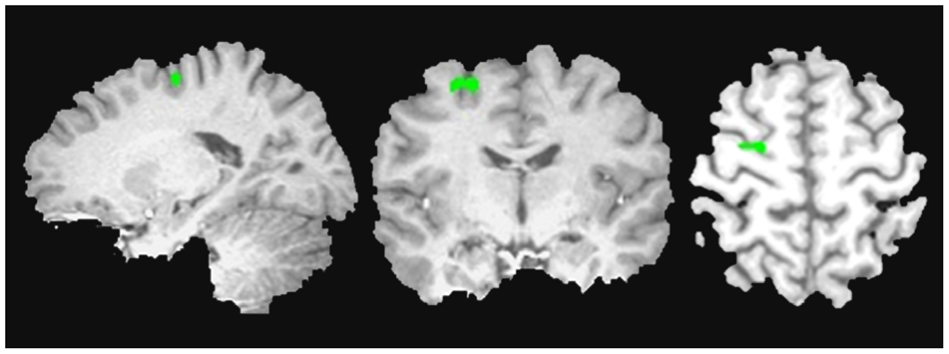
Region of interest analysis: TD vs. ADHD. Our ROI analysis utilizing a bilateral IFG and MFG mask revealed one region in the left MFG (BA 6) that was more active in the TD over the ADHD group during working memory.

The between-group ROI analysis revealed one region within the left MFG (BA 6), which was more active in the TD compared to the ADHD group (see Figure 5). This region overlapped with a region in the within-group TD contrast map.

#### 4.5. Whole-brain correlations between VSAT activation, RT and ADHD symptom ratings

In the TD group, correlations between RT and brain activation revealed 3 regions (see Table 5A.). The left pre-central gyrus extending into inferior/superior parietal lobe and right insula correlated positively with RT (more activation associated with longer RTs) and a negative correlation was observed for the right superior temporal gyrus extending into supramarginal gyrus. In the ADHD group, the correlation between brain activation and RT did not produce any regions. These results suggest brain activity is related to task performance for the comparison group, but this behavioral/functional coupling is absent in the ADHD group at this threshold.

**Table 5:** Whole-brain Correlations.

Region	Brodmann Area	Hem	Volume	Talairach coords. (center of mass)		
			(µl)	x	y	z
				(RL)	(AP)	(IS)
***A) TD Activation and Correct RT***						
IPL/SPL/preCG/postCG	1/3/4/7/40	L	1973	−25	−35	55
MTG/STG	22/37/39	R	598	44	−46	17
Insula/IPL	42	R	305	48	−29	20
***A) ADHD Activation and Inattentive Symptoms***						
IOG/MOG/Fusif Gyr/Declive	19/19/37	R	611	36	−66	−10

*Note:* Inattentive symptoms from the Conners' Parent Rating Scale Long Version; Fusif Gyr, fusiforn gyrus. All other abbreviations as Table 3 and 4. Corrected for multiple comparisons.

Correlations between brain activity and symptom severity identified a negative correlation between activity in the right inferior occipital gyrus extending into fusiform gyrus and the inattentive score (see Table 5B). Thus, children who were rated by their parents as having less severe attentional problems had increased activation in this region.

## Discussion

Working memory-related activity was evident in the TD control group in the bilateral MFG (BA 6), right MFG (BA 9) extending into ACC, pre-central gyrus, bilateral post-central gyrus (BA 4) and the right cingulate. The within-group contrast in the ADHD group failed to reveal any working memory-specific regions. The between-group contrast revealed regions in the bilateral insula, right claustrum, IFG, putamen and in the left medial FG. The ADHD group activated all of these regions significantly more than controls. Additional ROI-based analysis revealed a region in the left MFG that was more active in the TD group. Therefore, these regions were areas that significantly differed between groups in the working memory task.

TD participants activated a network of regions, including bilateral MFG, commonly activated in WM. The lateral PFC has been associated with working memory in a number of studies in adults [51], [52] and children [53] and has been implicated in top-down control or manipulation of information held in working memory [51], [52], [54], [55], [56]. Top-down attention processes refer to internal processes under conscious and effortful control, whereas bottom-up processes are usually automatically triggered by external events. In previous studies of working memory in typically developing individuals, the left lateral frontal cortex has been associated with encoding, maintenance and retrieval processes [54] or with selection of items from memory [57].

Although the ADHD group as a whole failed to demonstrate any significant activity in the regions defined by the TD group, the between-group working memory contrast failed to show significant group differences in these regions with the exception of the left MFG. Thus, left MFG was a working memory-related region that was hypoactive in the ADHD compared to the TD group. Hypoactivity in the frontal cortex during cognitive control is a largely consistent finding in ADHD [8], [58], [59], [60], [61], [62]. Impairment in frontal cortex, particularly in the left lateral PFC, may be related to the impairment ADHD individuals exhibit in storing task goals and rules, maintaining task set and re-establishing top-down attention processes, as this region has been implicated in these processes in TD adults [15], [47], [63], [64], [65]. A recent study suggested that working memory impairments in ADHD might result in inefficient task set maintenance in this group, reflected by decreased activation in lateral PFC [27]. Diminished activity in the left prefrontal cortex during working memory in boys with ADHD compared to control boys during an N-Back working memory task has been demonstrated [13]. Further evidence for the involvement of the left PFC in ADHD is documented in research showing thinner cortical thickness in this region is negatively correlated with symptom impairment in a study assessing long-term outcome in children with ADHD [24].

However, the lack of a between-group difference in the cingulate, motor regions and right MFG suggests that there was significant variability in these regions in the ADHD group, with some of the participants in the ADHD group activating these regions. In fact, correlations revealed that those ADHD subjects who successfully engaged bilateral MFG also displayed better performance. Two regions from the Main effect of Condition contrast overlapped with regions from the TD within-group map, namely the right MFG (BA 6) and the right precuneus, extending into post-central gyrus. Thus these regions are very likely to be engaged by both ADHD and TD groups during working memory processing.

The within-group contrast in the ADHD group did not result in working memory-specific regions, even though the behavioral data suggested that difficulty increased in both groups from MST to AT to VSAT tasks. Furthermore, the main effect of Group only revealed regions that were more active in the ADHD over TD group and thus are more active in general in the ADHD group. In fact, three regions, two in the right claustrum/insula and one in the left insula extending into the IFG, overlapped with the ADHD regions in the working memory between-groups contrast. This suggests that the ADHD group may be less likely to engage brain regions specifically for working memory or for more cognitively-demanding tasks in general. Similarly, in our previous working memory-imaging study in adults with ADHD, we found the ADHD group was more likely to engage the cerebellar vermis than controls, regardless of the task demands or whether or not they were actively taking a stimulant medication [14], [66]. Results from this task provide support for a lack of specificity in ADHD for task performance and brain activation that may be related to poor ability to modulate input from non-cognitive brain regions and hence output to external task demands [26].

Compared to the TD group, the ADHD group engaged the bilateral insula extending into the basal ganglia and the medial PFC. Activation in the insula has been noted in working memory encoding, maintenance and retrieval in TD adults [54], [67], [68] and during working memory in ADHD pediatric [28] and adult [30], [69] participants. Dickstein and colleagues' meta-analysis of 16 imaging studies of executive functioning in ADHD revealed that one of the areas that was likely to be hyperactive in participants with ADHD compared to controls was the insula (BA 13) [58]. Activation also extended to the claustrum, an area associated with sensorimotor integration. Hyperactivity in this region may be associated with increased processing of task irrelevant information. This may result in greater input from brain regions that feed into the claustrum, such as the motor, visual, auditory processing and executive regions and slower output to respond to task demands. As this activity was not specific to the VSAT, we expect that this region may be associated with more general processes rather than being associated with working memory *per se*.

Hyperactivity in the medial PFC in the ADHD compared to control group has previously been addressed in our examination of distractibility in ADHD, utilizing the same data set [33]. Briefly, hyperactivity in this region in ADHD during working memory is likely driven by increased *deactivation* of this area in the TD group. Medial PFC has been associated with the default mode attention network, which is usually deactive during cognitive demand. Failure to suppress activity in the default network during cognitive demand has been associated with errors in performance and distractibility not only in individuals with ADHD but also in healthy controls [33], [70], [71], [72]. We linked a failure to suppress activity, particularly in medial PFC, to increased RT variability in our previous study [33].

Whole-brain correlations revealed links between task performance and activation in the TD group in the middle and superior temporal lobes and in regions associated with working memory and attention in general, namely the inferior and superior parietal lobe. These correlations were absent in the ADHD group. Previous fMRI studies of working memory in ADHD found hypoactivity in parietal regions in adults with ADHD [10], [30] and in the temporal lobe of adolescents with ADHD [11] compared to the control group. Whole brain correlations also revealed a link between the BOLD signal in the right occipital region inattention such that children in the ADHD group with less severe inattention tended to activate this region more during the working memory paradigm. Right occipital lobe volume has been observed to be enlarged [73] and grey matter density increased [25] in children with ADHD. There is also evidence of hyper-perfusion and increased regional homogeneity in the occipital cortex in children with ADHD [74]. Perhaps these subjects were more likely to use visual strategies to perform the VSAT given that activation in the occipital gyrus is frequently associated with memory for visual information (e.g. [67], [75]).

The relatively small sample size utilized in this study not only limited our ability to test for age or gender effects but may also have compromised our ability to identify additional between-group differences. Between-group differences in the left MFG were only detectable using an ROI approach which is most likely due to a lack of power to detect this difference at a whole-brain level. Other limitations of this study include above average IQs for both groups that may limit the generalizability of the results and varying degrees of exposure to stimulant medication in ADHD participants. It is possible that exposure to medication altered brain activation, however recent studies comparing stimulant-naive and stimulant-exposed pediatric participants [76] challenge the extent to which these effects exist. The absence of a baseline condition with a lower cognitive demand than the MST, such as the fixation point, may have also benefited the analyses, but adding another condition would have extended the imaging session beyond a tolerable point for many of our younger children. Future studies should be designed to directly test whether individuals with ADHD use brain regions and circuits associated with motor, visual and tactile processing to compensate for underperforming brain regions when engaged in cognitive control or working memory paradigms.
